# Gene Expression Profile Signature of Aggressive Waldenström Macroglobulinemia with Chromosome 6q Deletion

**DOI:** 10.1155/2018/6728128

**Published:** 2018-10-04

**Authors:** Naohiro Sekiguchi, Junko Nomoto, Akihisa Nagata, Masahiro Kiyota, Ichiro Fukuda, Kazuaki Yamada, Naoki Takezako, Yukio Kobayashi

**Affiliations:** ^1^Division of Hematology, National Hospital Organization Disaster Medical Center, Tachikawa, Tokyo 190-0014, Japan; ^2^Department of Hematology, National Cancer Center Hospital, Tsukiji, Tokyo 104-0045, Japan; ^3^Division of Radiology, National Hospital Organization Disaster Medical Center, Tachikawa, Tokyo 190-0014, Japan; ^4^Division of Laboratory and Pathology, National Hospital Organization Disaster Medical Center, Tachikawa, Tokyo 190-0014, Japan

## Abstract

**Background:**

Waldenström macroglobulinemia (WM) is a rare, indolent B-cell lymphoma. Clinically, chromosome 6q deletion (6q del) including loss of the B lymphocyte-induced maturation protein 1 gene (BLIMP-1) is reported to be associated with poor prognosis. However, it remains unclear how the underlying biological mechanism contributes to the aggressiveness of WM with 6q del.

**Methods:**

Here, we conducted oligonucleotide microarray analysis to clarify the differences in gene expression between WM with and without 6q del. Gene ontology (GO) analysis was performed to identify the main pathways underlying differences in gene expression. Eight bone marrow formalin-fixed paraffin-embedded samples of WM were processed for interphase fluorescence in situ hybridization analysis, and three were shown to have 6q del.

**Results:**

GO analysis revealed significant terms including “lymphocyte activation” (corrected p value=6.68E-11), which included 31 probes. Moreover,* IL21R* and* JAK3* expression upregulation and activation of the B-cell receptor signaling (BCR) pathway including* CD79a*,* SYK*,* BLNK*,* PLCγ2*, and* CARD11* were detected in WM with 6q del compared with WM without 6q del.

**Conclusion:**

The present study suggested that the BCR signaling pathway and* IL21R *expression are activated in WM with 6q del. Moreover,* FOXP1 *and* CBLB* appear to act as positive regulators of the BCR signaling pathway. These findings might be attributed to the aggressiveness of the WM with 6q del expression signature.

## 1. Background

Waldenström macroglobulinemia (WM) is a rare, indolent type of B-cell lymphoma with monoclonal IgM gammopathy. B-cells, lymphoplasmacytic cells, and plasma cells (PCs) form neoplasms in WM, with the bone marrow (BM) being the main site of infiltration [1]. The disease has an overall annual incidence of three per million in the United States (US) [2, 3].

Cytogenetic analysis previously detected chromosome 6q deletion (del), including loss of the B-lymphocyte-induced maturation protein 1 gene (*BLIMP-1*) and* TNFAIP3/A20*, in 40%–55% of WM patients in the US and European countries [4–8]. Furthermore, this aberration is recognized as one of the factors associated with poor prognosis, higher serum IgM levels, and a higher International Prognostic Scoring System for WM (ISSWM) level [4–8].

However, it remains unclear how the underlying biological mechanism contributes to the observed aggressiveness of WM with 6q del. The present study therefore conducted an oligonucleotide microarray analysis to clarify the biological differences between WM with and without 6q del.

## 2. Methods

### 2.1. Patients

Newly diagnosed symptomatic WM patients attending the National Hospital Organization Disaster Medical Center from January 2010 to March 2014 were enrolled in this study. Samples of cases whose comprehensive agreement had been obtained were subject for the study. The WM diagnostic criteria used in the present study were according to the revised 4^th^ edition of World Health Organization (WHO) classification of tumours of haematopoietic and lymphoid tissues [1]. Briefly, these included detection of the IgM monoclonal protein and BM neoplastic cells including lymphocytes, CD20-positive lymphoplasmacytic cells, and some PCs more than 10 % and presence of symptoms.

Patient clinical data were obtained from their medical charts and included age, sex, performance status (PS), hemoglobin level, platelet count, serum *β*2-microglobulin level, ISSWM [9], serum M-protein, B symptoms, hepatomegaly, splenomegaly, and lymphadenopathy. PS was determined according to the Eastern Cooperative Oncology Group scale [10].

### 2.2. Pathological Review

A pathological review confirmed that all cases were IgM-WM according to the revised 4th edition of WHO classification [1]. MALT lymphoma or CLL with monoclonal IgM protein were carefully excluded from the study. Using BM biopsied specimens, the infiltration pattern of neoplastic cells was divided into four groups: diffuse, interstitial, paratrabecular, and nodular. Additionally, the PC%, lymphoplasmacytic cell %, lymphocyte %, and total neoplastic cells% in BM were evaluated from BM smears.

### 2.3. Fluorescence In Situ Hybridization Analyses (FISH)

Cytogenetic aberrations were obtained by FISH analysis using the A20/BLIMP-1/SHGC-79576 Three Color Probe (Cancer Genetics Italia™ SRI, Mila, Italy) to detect 6q del.* A20 *and* BLIMP-1 *are located at 6q23 and 6q21, respectively, and the 6q deletion can be easily detected by comparing the signal intensity of this region with that of the chromosome 6 centromere [11]. Interphase FISH was performed using stored BM aspirate patient specimens, and results were recorded according to ISCN 2016 [12]. The normal cut-off values for deletions in the chromosomal region were defined as means + 3SD of the results for 20 normal controls.

### 2.4. Oligonucleotide Microarray Analyses

cDNA microarray analysis was performed following the manufacture's instruction. Briefly, formalin-fixed paraffin-embedded BM biopsied samples were deparaffinized and total RNA was extracted using the RNeasy FFPE kit (Qiagen, Venlo, the Netherlands). Isolated total RNA was converted into cDNA, followed by cDNA amplification. After this, 4 *μ*g of cDNA was fragmented at 37°C for 30 min and then biotinylated using the Encore cDNA (Nugen, San Carlos, CA). Biotin-labeled cDNA was hybridized to the Human Genome U133 Plus 2.0 Array (Affymetrix, Santa Clara, CA). The array was incubated for 18 h at 45°C, then automatically washed, and stained using the GeneChip Hybridization, Wash, and Stain Kit (Affymetrix). The array was scanned using a GeneChip Scanner 3000 7G.

### 2.5. Immunohistochemistry

Paraffin-embedded BM specimens underwent heat-induced antigen retrieval in pH 6.0 citrate buffer. Polyclonal antibodies against IL-21R (1:500, NBP1-87502, Novus Biological, Littleton, CO) were used. After incubation with the primary antibody, slides were incubated with the secondary antibody, One-Step Polymer-HRP (BioGenex, Fremont, CA), for visualization. The IL-21R staining pattern was categorized into three groups: <30%, 30%–70%, and >70% of lymphoplasmacytic cells and PCs.

### 2.6. Statistical Analysis

Descriptive statistics of each clinical data were calculated by IBM SPSS Statistics for Windows 24.0J. Oligonucleotide microarray analyses were carried out using GeneSpring GX software version 13.0 (Agilent Technologies, Santa Clara, CA). Briefly, described as follows, the expression values were calculated from the raw data using the MBEI algorithm [13]. Only probes that signal intensity were within 20 to 100 percentile in each array which were included in the comparative analysis. The genes corresponding to probes having a change in intensity exceeding a ratio of four were considered as genes with a significant differential expression pattern. In parallel, unpaired t-test with unequal variance (Welch's t-test) was performed to compare the means of the two groups of replicates. Probes with a p value less than 0.05 were considered having significant different signal value. GO analysis was then performed on the sets of probes above the 4-fold ratio that passed the t-test to identify the main pathways underlying differences in gene expression. We arbitrary selected 4-fold different expression as a cut-off ratio instead of considering false discovery rate, because of the small sample size, and we performed the oligonucleotide microarray analysis as an exploratory research. Ontologies with corrected p-values <0.1 were considered significant. The microarray datasets used in the present study were deposited in the Gene Expression Omnibus under accession number GSE70511 (http://www.ncbi.nlm.nih.gov/geo/query/acc.cgi?acc=GSE70511).

## 3. Results

### 3.1. Clinical Features

Eight patients were enrolled in the study, and characteristics are shown in Table 1. Briefly, the median age was 71.5 years, and the male/female ratio was 5/3. ISSWM low/int/high was observed as 0/4/4. Median monoclonal M-protein levels were 2.65 g/dL (range: 0.8–4.28 g/dL). A total of two patients were observed to have constitutional B symptoms; one had hepatomegaly, and another had splenomegaly. Adenopathy and cold agglutinin disease were found in two patients each. Diffuse, interstitial, paratrabecular, and nodular patterns of neoplastic cell invasion into the BM were seen in seven, zero, one, and zero patients, respectively. The median small lymphocyte %, lymphoplasmacytic cell %, and PC% were 39.0 %, 3.3%, and 1.9 %, respectively, with total neoplastic cells accounting for 45.7%.

### 3.2. Cytogenetic Analysis and Correlations between 6q del and Individual Characteristics

**Figure 1:**
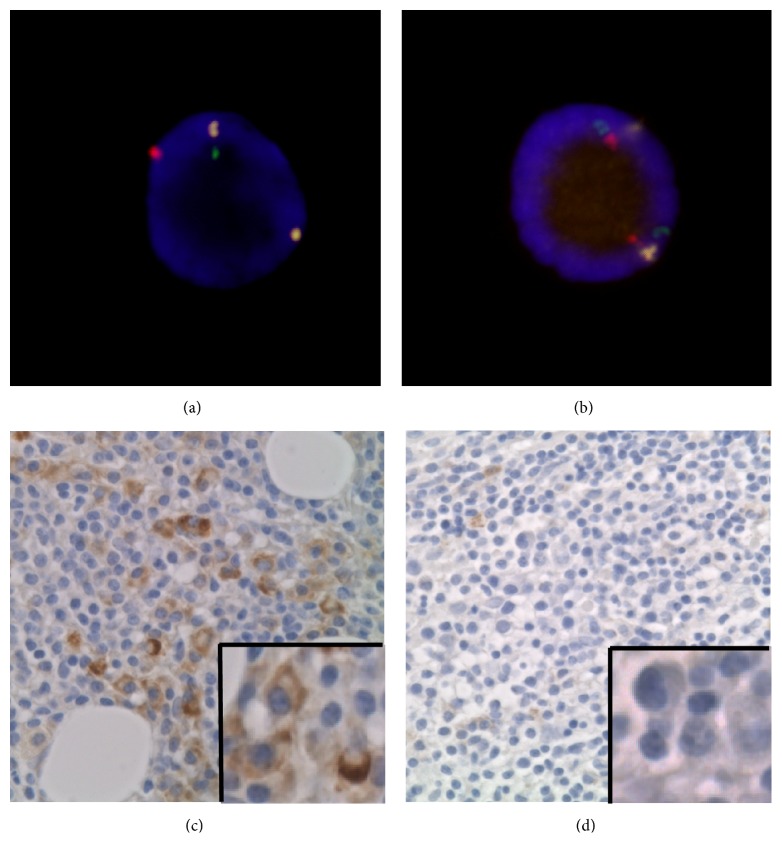
**FISH analysis of BM aspirates using the A20/BLIMP1/SHGC-79576 Three Color Probe (Cancer Genetics Italia™) and IL21R staining of a BM biopsied specimen.** Green, red, and yellow signals represent BLIMP1, A20, and SHGC-79576, respectively. (a) 6q del pattern. One green, one red, and two yellow signals were detected, showing nuc ish(SHGC-79576 x2, BLIMP-1x1, A20 x1). (b) Normal pattern. Two green, two red, and two yellow signals are recognized, showing nuc ish (SHGC-79576, BLIMP-1, A20) x2. (c) >70% positivity of IL-21R in lymphoplasmacytic cells and plasma cells (original magnification ×600). (d) <30% positivity of IL-21R in lymphoplasmacytic cells and plasma cells (original magnification ×600).

To detect 6q del, fluorescent in situ hybridization (FISH) analysis was performed (Figures 1(a) and 1(b)). The cut-off value for loss of* BLIMP-1* and* A20* was 3%. Three of the eight cases had 6q del, in which both genes were deleted, and the other five cases did not have 6q del. Median serum M-protein levels were 3.14 g/dL in patients with 6q del, compared with 2.25 g/dL in those without 6q del. ISSWM low/int/high was 0/1/2 in patients with 6q del, compared with 0/3/2 in patients without 6q del. The median small lymphocyte %, lymphoplasmacytic cell %, PC %, and total neoplastic cell % were 41.6 %, 7.1%, 2.0%, and 46.4%, respectively, in those with 6q del, versus 36.4%, 3.0%, 1.8%, and 41.4%, respectively, in those without 6q del.

### 3.3. Oligonucleotide Microarray

All the eight cases' samples were subjected for oligonucleotide microarray analysis. To clarify the differences in gene expression between WM with and without 6q del, we performed oligonucleotide microarray analyses and detected that a total of 428 probes, corresponding to 324 annotated genes, were upregulated (p value < 0.05 and fold change > 4.0), and a total of 112 probes, corresponding to 96 annotated genes, were downregulated (p value < 0.05 and fold change < 0.25) in WM with 6q del (Supplementary Tables 1 and 2). Among genes located on chr 6q, 4 genes were upregulated, and 5 genes were downregulated, while both* BLIMP-1* and* A20* were neither upregulated nor downregulated.

Consecutively, gene ontology (GO) analysis was performed, and GO terms including “lymphocyte activation” (corrected p value=6.68E-11) and “B-cell activation” (corrected p value=2.15E-08) were statistically significant in the upregulated gene lists relative to the all genes on the microarray (Table 2). A network analysis of GO terms associated with “lymphocyte activation” included 31 probes and 30 genes, listed in Table 3. Genes involved in the B-cell receptor (BCR) signaling pathway, including* CD79a*,* SYK*,* BLNK*,* PLCγ2*, and* CARD11*, were shown to be activated in WM with 6q del compared with WM without 6q del. Other upregulated genes included* IL-21R*,* JAK3*,* IFNγ*, and* FOXP1*. In contrast, the GO term “plasma cell differentiation” was not statistically significant (data not shown).

### 3.4. Immunohistochemistry of Interleukin (IL)-21R

To confirm the upregulation of the IL21R protein in WM with 6q del, we performed immunostaining analysis. Figures 1(c) and 1(d) show that all three patients with 6q del had >70% positivity of IL-21R in lymphoplasmacytic cells and PCs. In WM without 6q del, one case was categorized as 30%–70% positivity, while the remaining four cases showed <30% positivity.

## 4. Discussion

WM is a rare entity of low-grade B-cell lymphoma with IgM monoclonal gammopathy according to classification by the WHO [1]. The most commonly involved site of infiltration is the BM, and some patients have adenopathy, hepatomegaly, and splenomegaly [1]. Neoplastic cells consist of CD20-positive B-cells and lymphoplasmacytic cells, while some PCs are also considered to be neoplastic [14]. Recently, Treon et al. [15] reported that the* MYD88* L265P mutation has been recognized in about 90% of WM cases in the US and in 70%–90% of cases worldwide [16]. This mutation is also found in 47% of patients with IgM monoclonal gammopathy of undetermined significance (IgM-MGUS) and is associated with a higher risk of disease progression to WM and splenic marginal zone lymphoma [17]. It is known to be a major mechanism of oncogenesis and is linked with Toll-like receptor (TLR) signaling pathway activation [15]. Furthermore, Yang et al. identified Bruton tyrosine kinase (BTK) complexed to MYD88 in L265P-expressing WM cells, with preferential binding of MYD88 to phosphorylated BTK [18].

In contrast, 6q del, including loss of* BLIMP-1* [19–21] and* A20* [22, 23], is recognized in 40%–55% of WM cases in the US and European countries [4–8, 14]. However, it is not recognized in IgM-MGUS, suggesting that it is a secondary event [7]. 6q del has long been thought to be involved in oncogenesis and is also reported to be associated with poor prognostic factors, such as higher levels of IgM and C-reactive protein and poor-risk patients with high ISSWM levels [4–6]. Nevertheless, the underlying biological mechanism of 6q del in WM is unclear.

Several studies regarding genes expression analyses in WM were performed, and these studies contributed to elucidate the biology and activated pathway in WM [24–26]. Chng et al. firstly conducted genes expression analyses in WM [24]. They reported that the most significantly upregulated gene was* IL6*, and the most significantly associated pathway for genes-set was MAPK signaling in WM compared to chronic lymphocytic leukemia (CLL) and multiple myeloma (MM). San Miguel and colleagues found deregulation of genes involved in plasma cell differentiation including* PAX5*, which was overexpressed, while* BLIMP-1 and IRF4* were underexpressed in WM in comparison with CLL and MM. In addition, they hypothesized that lack of* PAX5* repression contributed to the upregulation of 3 genes of BCR signaling pathway including* CD79, BLNK, *and* SYK* in WM [25]. Jiménez et al. reported that* CD79A* (B cell activation),* IRF3*,* MYD88*,* MEK1*,* P38* (TLR pathway), and WNK1 (MAPK pathway) were overexpressed in WM compared to IgM-MGUS, in which pathways might be responsible for WM cell growth and survival [26].

One of the unique findings of the present study was that genes involved in the BCR signaling pathway [23], including* CD79a*,* SYK*,* BLNK*,* PLCγ2*, and* CARD11* are upregulated in WM with 6q del patients compared with those without 6q del. Little is known about BCR pathway upregulation in WM [25], although various subtypes of B-cell lymphomas are associated with BCR pathway activation [27]. However, Argyropoulos et al. suggested that the BCR pathway is activated in WM following phosphoprofiling analysis [28]. Moreover, it is widely accepted that* BLIMP-1* suppresses B-cell proliferation and activation, including the BCR signaling pathway, and orchestrates mature PC differentiation by suppressing the expression of genes necessary for commitment and maintenance of the B-cell identity, including* PAX5* and* XBP-1* [29, 30].

**Figure 2:**
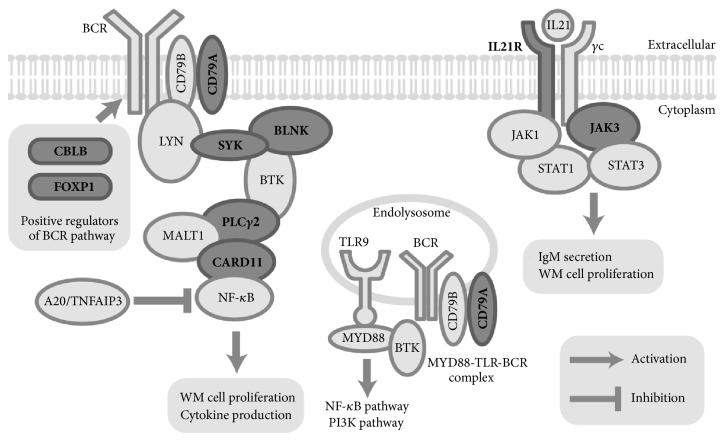
**Schema of putative biological mechanisms of aggressiveness in Waldenström macroglobulinemia with 6q deletion.** Upregulated genes in the present study are represented by bold font and dark shapes.

During preparation for this manuscript, Staudt and colleague reported that MYD88, TLR9, and BCR complexes (My-T-BCR Complexes) exist in activated B-cell like diffuse large B-cell lymphoma and in WM, which might play a role in tumor growth and survival [31] (Figure 2). In the present study, the BCR signaling pathway was activated in WM with 6q del patients, which might result from the decreased inhibition occurring through the loss of* BLIMP-1*. However, no difference in the proportion of PCs in the BM was observed between WM with and without 6q deletion. Furthermore,* BLIMP-1 *was not among downregulated genes in the present study, suggesting that loss of* BLIMP-1* does not affect PC differentiation. Considering these results, the loss of* BLIMP-1* did not appear to act as a loss of heterozygosity regarding PC differentiation.

We also observed* IL21R* and* JAK3* overexpression in WM with del 6q. Investigators from the Mayo Clinic previously reported that the IL21/21R pathway contributes to IgM secretion and WM cell proliferation via the JAK/STAT signaling pathway in a WM cell line and WM patient samples [32]. Considering these results, it is conceivable that increased IgM levels in WM with 6q del are attributable to IL21/21R pathway activation. In the present cohort, the median M-protein level was higher in patients with 6q del than those without 6q del, although the result was not statistically significant because of the small sample size.

Our cDNA microarray analysis of WM with 6q del. also revealed the overexpression of FOXP1 and the Casitas B-lineage lymphoma b gene (CBLB).* FOXP1 *overexpression is widely accepted to be a factor of poor prognosis in activated B-cell-like subtype diffuse large B-cell lymphoma and marginal zone B-cell lymphoma, mucosa-associated lymphoid tissue type [33, 34]. Its overexpression was also shown to lead to the constitutive activation of the nuclear factor-*κ*B pathway as well as BCR, CD40, and TLR signaling pathways [35].* CBLB* encodes a protein involved in the ligand-induced clustering of BCR on the cell surface and delivery of BCR-captured ligands to TLR9 [36]. Considering these findings,* FOXP1* and* CBLB* might act as positive regulators for the BCR signaling pathway in WM with 6q del. patients (Figure 2).

Our present findings might represent an aggressive expression signature of WM with 6q del., although it should be noted that the study was rather exploratory and the sample size was very small.

## 5. Conclusion

The present study suggested that the BCR signaling pathway and* IL21R *expression are activated in WM with 6q del., and* FOXP1 *and* CBLB* appear to act as positive regulators of the BCR signaling pathway. Thus, our present findings might represent an aggressive expression signature of WM with 6q del.

## Figures and Tables

**Table 1:** Patient characteristics.

	all (N=8)	6q del (+) (N=3)	6q del (-) (N=5)
Median Age (old, range)	71.5 (58-79)	76 (70-79)	71 (58-76)

Sex (M/F) (%)	5/3	2/1	3/2

PS>1 (%)	5	2	3

Hb <11.5 g/dL (%)	6	3	3

plt <10×10^9^/L (%)	1	0	1

*β*2MG>3mg/L (%)	3 (64.3)	2	2

Median M-protein (g/dL)	2.65 (0.8-4.28)	3.14 (2.13-4.28)	2.25 (0.8-3.87)

ISSWM(Low/Int/High) (%)	0/4/4 (9/36/55)	0/1/2	0/3/2

B-symptom (%)	2 (29.4)	1	1

Hepatomegaly (%)	1 (23.8)	0	1

Splenomegaly (%)	1 (23.8)	0	1

Lymphadenopathy (%)	2(25)	0	2

Cold agglutinin disease	2	0	2

Infiltration pattern (D/P)	7/1	3/0	4/1

Plasma cell (%)	1.9 (1-3.6)	2.0 (1-2.2)	1.8 (1.2-3.6)

Lymphoplasmacytic cell (%)	3.3 (2.4-8.6)	7.1 (2.6-8.6)	3.0 (2.4-5.2)

Small lymphocyte (%)	39.0 (29.4-62.8)	41.6 (35.9-62.8)	36.4 (29.4-46.8)

Total neoplastic cell (%)	45.7 (36-72.4)	46.4 (45-72.4)	41.4 (36-50.4)

PS: performance status; Hb: hemoglobin; plt: platelets; *β*2MG: *β*2-microglobulin; cCa: calculated Ca; ISSWM: International Prognostic Scoring System for Waldenström Macroglobulinemia.

**Table 2:** Significant terms on the gene ontology list.

GO Number	GO term	P-value	Numbers of probes
GO:0046649	lymphocyte activation	6.68E-11	31

GO:0043486	histone exchange	1.81E-10	14

GO:0034508	centromere complex assembly	3.22E-10	14

GO:0043044	ATP-dependent chromatin remodeling	1.02E-09	14

GO:0045321	leukocyte activation	2.49E-09	32

GO:1903706	regulation of hemopoiesis	9.44E-09	27

GO:0042113	B cell activation	2.15E-08	18

GO, gene ontology.

**Table 3:** Genes up-regulated in WM with 6q del compared with WM without 6q del.

Function categories	Up-regulated gene
B-cell receptor signaling pathway	*CD79a, SYK, BLNK, PLCγ2, CARD11*

IL21/21R signaling pathway	*IL21R, JAK3*

NF-kB activater	*FOXP1*

Ubiqutin ligase	*CBLB*

Ikaros zinc finger family	*IFZF3*

Cytokines	*IFNγ*

Other genes	*MSH6, IMPDH2, AKAP17A, CCR7, MEF2C, POU2F2, ITPKB, BANK1, IL7R, LAX1, ERCC1, PRKCB, KLRC4-KLRK, HDAC9, ITGAL, GON4L, PSEN1, RHOH, PSEN1*

WM, Waldenstrom macroglobulinemia.

## Data Availability

The microarray data sets used in the present study were deposited in the Gene Expression Omnibus under accession number GSE70511 (http://www.ncbi.nlm.nih.gov/geo/query/acc.cgi?acc=GSE70511).

## Supplementary Materials

Supplementary Materials
** Supplementary Table** **1.** Upregulated gene sets in WM with 6q del compared with WM without 6q del. The sheet contains 428 probe sets. Among them, the first 342 probes that were allocated to genes or loci are shown according to Entrez Gene number in ascending order, and the remaining 86 probes that were not allocated to loci are shown according to fold changes in descending order.** Supplementary Table ****2.** Downregulated gene sets in WM with 6q del compared with WM without 6q del. The sheet contains 112 probe sets. Among them, the first 98 probes that were allocated to genes or loci are shown according to Entrez Gene number in ascending order, and the remaining 14 probes that were not allocated to loci are shown according to fold changes in ascending order.
